# Improving generalist end of life care: national consultation with practitioners, commissioners, academics, and service user groups

**DOI:** 10.1136/bmj.a1720

**Published:** 2008-10-01

**Authors:** Cathy Shipman, Marjolein Gysels, Patrick White, Allison Worth, Scott A Murray, Stephen Barclay, Sarah Forrest, Jonathan Shepherd, Jeremy Dale, Steve Dewar, Marilyn Peters, Suzanne White, Alison Richardson, Karl Lorenz, Jonathan Koffman, Irene J Higginson

**Affiliations:** 1 King’s College London, Department of Palliative Care, Policy and Rehabilitation, Weston Education Centre, London SE5 9RJ; 2 King’s College London, Department of General Practice and Primary Care, London SE11 6SP; 3 Primary Palliative Care Research Group, University of Edinburgh, Edinburgh EH8 9DX; 4 General Practice and Primary Care Research Unit, Department of Public Health and Primary Care, University of Cambridge, Cambridge CB2 2SR; 5 Centre for Primary Health Care Studies, Warwick Medical School, University of Warwick, Coventry CV4 7AL; 6 King’s Fund, London W1G 0AN; 7 King’s College London, Florence Nightingale School of Nursing and Midwifery, London SE1 8WA; 8 VA Greater Los Angeles Healthcare System, School of Medicine, University of California, Los Angeles, CA 90073, USA

## Abstract

**Objective** To identify major concerns of national and local importance in the provision, commissioning, research, and use of generalist end of life care.

**Design** A national consultation and prioritising exercise using a modified form of the nominal group technique.

**Participants** Healthcare practitioners, commissioners, academics, and representatives of user and voluntary groups.

**Setting** Primary and secondary care, specialist palliative care, and academic and voluntary sectors in England and Scotland.

**Results** 74% of those invited (210/285) participated. The stage of life to which “end of life care” referred was not understood in a uniform way. Perceptions ranged from a period of more than a year to the last few days of life. Prominent concerns included difficulties in prognosis and the availability of adequate support for patients with advanced non-malignant disease. Generalists in both primary and secondary care were usually caring for only a few patients approaching the end of life at any one time at a point in time. It was therefore challenging to maintain skills and expertise particularly as educational opportunities were often limited. End of life care took place among many other competing and incentivised activities for general practitioners in the community. More needs to be known about models of end of life care and how these can be integrated in a generalist’s workload. A greater evidence base is needed about the effectiveness and application of current tools such as the gold standards framework and Liverpool care pathway and about models of palliation in patients with diseases other than cancer.

**Conclusions** Definitions of end of life care need clarification and standardisation. A greater evidence base is needed to define models of good practice together with a commitment to provide education and training and adequate resources for service provision. More needs to be known about the context of provision and the influence of competing priorities and incentives.

## Introduction

Most “end of life care” occurs in a generalist setting rather than a specialist palliative care setting.^1^ ^2^ ^3^ ^4^ Worldwide most people spend most of their last year of life at home being cared for by family, family doctors, community nurses, and as outpatients by hospital clinicians^1^ ^5^ ^6^ ^7^ ^8^ ^9^ ^10^ and often with social care support. In industrialised countries, however, most people will die in institutions such as hospitals, nursing homes, and care homes, where generalists provide most care. A minority will die in their own homes, although the specific proportions vary according to the structures of different national healthcare systems. 3 11 12 13 14 15 16 17 Little is known about the effectiveness of different models of care delivery or about the underlying issues of concern that inform policy and service development. Over the past two decades, however, research documenting the experiences of poor control of symptoms in patients with advanced disease has fuelled the development of specialist palliative care services and the support they provide to generalists. 18 19 20 Patients’ choice has become a feature of government policy in several countries, but there is little evidence of an impact on the place of care and of death.^21^ ^22^

Generalist end of life care has recently become a major focus of health policy in the United Kingdom.^23^ ^24^ ^25^ ^26^ ^27^ ^28^ Many initiatives are being developed to improve care. In England in July 2008, the Department of Health published an End of Life Care Strategy to “bring about a step change in access to high quality care for all people approaching the end of life” in all care settings. This is to be achieved with a whole systems and care pathway approach for commissioning and providing integrated services, improving coordination. It will involve workforce development including education and training for generalists as well as specialists. Other areas include developing care plans, enabling rapid access to care and enhancing support to carers. It also identifies the need for improved measures of service provision, enhanced research, and funding.^28^ This strategy builds on the end of life care programme, which extended the uptake of tools such as the gold standards framework (a framework to assess needs and preferences, plan care, and improve communication in primary care, www.goldstandardsframework.nhs.uk), the Liverpool care pathway (a document recording care provided to patients in the last days of life, www.mcpcil.org.uk/liverpool_care_pathway), and the preferred priorities of care (a document held by patients that records preferences for care and service use at the end of life, www.cancerlancashire.org.uk/ppc.html).

A review of health services in London identified a lack of adequate planning for end of life care at the individual, organisation, and system levels, concerns for standards of care of the dying in hospital, and the variable quality in end of life care experienced (www.healthcareforlondon.nhs.uk/background.asp). These views have been echoed by professional bodies such as the Royal College of Physicians.^29^ ^30^ There is limited evidence from research to support the conclusions and recommendations arising from these reviews and reports and lack of research has been recognised by the Department of Health.^28^

As part of a scoping exercise to determine research priorities in generalist end of life care^31^ ^32^ ^33^ we investigated what was understood by generalist end of life care and the current concerns and preferences for service research and development from the perspectives of clinicians, user groups, commissioners, academics, and policy makers.

## Methods

*Design*—We undertook a national consultation and prioritisation exercise using a modified form of the nominal group technique^34^ in London, the east of England, Warwickshire, and Scotland and with representatives of English national organisations.^31^ ^32^ ^33^ Local research teams were established in each area, and the London team conducted the English national consultation. The consultation exercise was undertaken over seven months.

*Participants*—We invited health and social care practitioners from primary, secondary, and tertiary services and from specialist palliative care, service commissioners, policy makers, academics, and user and voluntary groups to participate.^31^ ^32^ ^33^ Participants were selected on a purposive basis from among major professional and academic organisations and user groups, those known to the project teams, and individuals prominent in end of life care, to gain wide representation from among the different stakeholder groups.

*Data collection*—We used short semistructured questionnaires standardised across all five consultations. These were developed by the research teams, the project advisory group, and grant collaborators and piloted. Interviews were usually undertaken by telephone but occasionally took place face to face. A shortened version of the questionnaire was sent by email. Informants were offered a choice of method of response. Questions included views about the generalist’s role, specific local concerns, access, education, training, and support (box 1). We defined “generalist end of life care” as care provided by health or social care professionals other than those whose remit was specialist palliative care. We proposed that “end of life care” encompassed care provided within the last year(s) of life to anyone with an advanced progressive disease that was likely to shorten their life. We invited participants to challenge these definitions. Each team identified a local researcher to undertake recruitment, data collection, and analysis according to an agreed protocol.

**Box 1 Topics from the question schedules used for interviews and email questionnaires**

- What do you understand by the term “end of life care”?
- What is the generalist’s role in providing end of life care and how does this contrast with the specialist palliative care role?
- Are there any specific concerns about generalist palliative care in your area relating to:
  1. Knowledge and expertise
  2. Working with other agencies
  3. Availability of specialist support
  4. Care homes
  5. Cost effectiveness
  6. Continuity of care including out of hours care?
- Are there issues of inequitable access to care in your area?
- What more needs to be done in terms of:
  1. Education and training for generalists
  2. Providing more support for patients and families
  3. Providing more support for generalists?
- What research would be most useful in supporting better generalist palliative care?

*Nominal group technique*—The nominal group technique is a method for generating consensus and involves seeking views, discussing and clarifying issues, and voting on priorities.^34^ We conducted stages as outlined in the figure[Fig fig1]. In each of the local and national settings we held a consultation meeting to present findings from the questionnaires and participants discussed and clarified issues. We modified the method to generate ideas before the meetings and to allow those unable to attend to participate by email or telephone. Voting was undertaken to determine research priorities (reported elsewhere).^31^ ^32^ ^33^

**Figure fig1:**
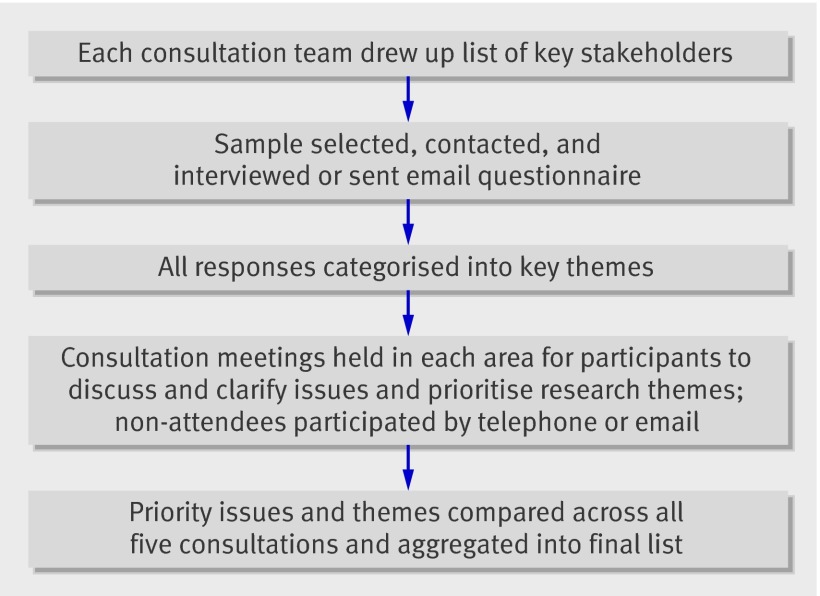
Key stages undertaken in consultation process by each of five nominal groups

*Data analysis*—Each local research team undertook a thematic analysis of participants’ responses by reviewing interview transcripts and identifying key themes and categories. Key themes were then discussed by all research teams and a common core of categories agreed to enable comparison together with themes specific to each locality. Participants’ responses were grouped under these themes and were presented back to participants at each consultation meeting, providing attendees with an opportunity for discussion and clarification. The results from each of the five consultations were synthesised to identify widely shared issues as well as local priorities. This was undertaken by the coordinating team in discussion with the other teams. A consensus was finally achieved. The analysis was undertaken at the same time as a parallel analysis to identify research priorities for the project funders (reported elsewhere).^31^ ^32^ ^33^

*Rigour*—To ensure rigour and quality control across the five consultations, all research teams held regular teleconference meetings to discuss progress and refine the common protocol during data collection and analysis. The analysis was completed by drawing on the expertise within the research teams and the project advisory group. The project coordinator circulated results for each stage of the process to enable discussion, debate, and agreement of the final categories.

## Results

### Responses to the consultation

Of the 285 participants invited, 210 (74%) responded, including commissioners and policy makers (17/33), generalist clinicians and practitioners (doctors, nurses, and ambulance and social service personnel) (58/81), members of voluntary sector/user groups (49/58), specialist palliative care clinicians (51/58), academics (23/29), and others such as managers of cancer networks (12/26). Participants were recruited from English national organisations (49/71), locally in London (50/65), the east of England (26/34), and Warwickshire (25/38) and both nationally and locally in Scotland (60/77). Participants expressed considerable enthusiasm, with most suggesting that generalist end of life care was a vital but neglected issue about which little was known.

### Defining generalist end of life care

There was little consensus about what end of life care and generalist meant. End of life care had different meanings for different respondents (box 2). For some, it was the time from diagnosis of a condition that would probably result in death. For others it comprised a period of time after that diagnosis, which could be a year or more or the last days of life.

**Box 2 Definitions of generalist end of life care**

**Definitions of generalist**

“Anyone who isn’t a specialist in palliative care” (specialist in palliative care)
“Most practitioners who do not work in specialist palliative care are specialists in other areas of practice rather than generalists, so, for example, I think of district nurses as being specialist community nurses by virtue of their specialist recordable qualification” (specialist in palliative care)
“It’s not just a dichotomy of generalist and specialist. Among all staff there are levels of experience and training. Some experienced generalists may have greater knowledge of palliative care principles and issues than more recently practising specialists” (generalist)
“I am thinking of generalist in two ways. Firstly there is the generalist doctor or nurse—eg, the GP or community nurse. But then there is generalist palliative care provided by specialists in another field—eg, a consultant physician providing good end of life care” (generalist)

**Definitions of end of life care**

“I don’t think of ‘end of life’ as any specific time but rather the phase from when it is clear that somebody is going to die in the foreseeable future until the end of their life” (academic)
“I believe ‘end of life care’ is a term that encompasses palliative and terminal phases” (generalist)
“I understand it to mean the last few days of life, although the term is generally not well defined and used differently according to different speakers and contexts” (generalist)
“End of life care is care of an ill person who may be within two years of death” (generalist)
“My general understanding of end of life care is that it refers specifically to the last stages of a progressive disease/condition that will end in death . . . My experience of ‘reality’ is that when people talk about end of life care, I assume they are talking mainly about people with cancer” (academic)

Generalists included all those working in health and social care in acute, rehabilitation, and continuing care settings in nursing and residential homes. For some, they included general practitioners, district nurses, and geriatricians, although all could be considered to be specialists in their own discipline (see box 2). Generalists were seen to deal with all conditions on a daily basis and their roles included coordination of care, key worker, gatekeeper, and referrer to others, particularly specialist palliative care services.

Generalist end of life care could be more concerned with non-malignant disease compared with specialist palliative care, which was seen to be largely concerned with cancer. Because of difficulties in identifying end of life in non-malignant disease, end of life care was also thought to be biased towards cancer, whether in a generalist or specialist palliative care setting. Such differences in understanding seemed to affect the perceived relevance of referral to, or awareness of, service options. Variability of standards of generalist end of life care was a major concern among all groups.

### Skills and expertise in palliative care

Within the NHS, capacity to provide palliative care in a generalist setting was seen by generalists and specialists alike to depend on the balance of team composition, team skills, and access to specialist support. Acquisition and maintenance of skills in palliative care was thought to be difficult for generalists as they usually cared for relatively few people nearing the end of life. All groups expressed concerns about the variability of skills in palliative care and identified education and training as key issues. Reaching the “disengaged generalist” and developing skills was thought to be difficult when educational opportunities were limited (box 3).

**Box 3 Developing and maintaining the skills of generalists and obstacles to skill development**

**Engaging busy practitioners in education and training**

“How do you get hold of the people who don’t come? We get good attendance but the attendance we get is probably the same people and so we’re not necessarily reaching the people who need the education most” (manager)

**Funding constraints**

“Although there is education to support generalists to improve their knowledge and experience, this is not always supported in practice and more recently in our local area we have had a palliative care education course cancelled, as funding has been a concern . . . education and follow-up support is paramount in order to support the generalist workforce” (generalist)

**Education about the needs of carers**

“A major problem is that carers seem to remain ‘invisible’ to many healthcare professionals . . . Education and training for generalists should always include coverage of the role, issues . . . .and not to forget the statutory rights of carers and the generalist’s responsibility to ensure that these are properly dealt with” (voluntary group)

**Turnover of staff**

“People with dementia who are living at home often have a huge turnover in the domiciliary care staff that are supporting them. This means there is no opportunity for a relationship to develop and for the care staff to build expertise in understanding and supporting that individual. Turnover in many care homes is often high, which also compromises the quality of care” (voluntary group)

**Appropriate skill mix**

“There are problems in that the number of community nurses at ‘sister’ level appears to have been reduced and inadequate recognition is given to their role. More emphasis on the importance of palliative care as part of primary care would be helpful, even if this means specific financial awards for providing it, similar to the financial awards given to GPs for other initiatives such as blood pressure control, etc” (generalist)

All groups were concerned that in some geographical areas, lack of skilled professionals and social carers limited the quality and quantity of good care that could be delivered, often because of poor recruitment and retention of staff. Access was further thought to be threatened by the reorganisation of district nursing teams on a case basis rather than on a geographical basis, which might have broken important links with general practices. The transfer of some district nurses into community matron roles was reported to have left gaps in provision. In care homes the right skill mix was considered an important issue as well as ensuring that perceptions of status did not preclude care assistants from participating in case discussions. Most groups of participants were concerned about perceived lack of awareness of approaches to end of life care in care homes.

### Place, organisation, and models of care

*Place of care and death*—Participants highlighted the mismatch between patients’ preferences and their actual place of care and death. Among those who voted for research priorities, nearly half (78/167) thought a better understanding was needed about patients’ experiences of hospital, home, and care homes and about resources needed to provide support at home. They thought that more attention also needed to be paid to care in hospitals.

*Generalist models*—Generalist models of end of life care, both in primary and secondary care settings, were a priority for development. Important gaps in knowledge about the impact of end of life care on caseloads and about the ways generalists and palliative care specialists can best work together were also noted.

*Out of hours and continuity of care*—Many participants were concerned about the need to improve primary out of hours care at the end of life, and almost half (80/167) considered this a research priority (box 4). Examples were reported of patients having to contact emergency services inappropriately where no out of hours district nursing services were provided. Transfer of information between out of hours organisations about patients at the end of life was seen to be less than optimal. Continuity of care was further reported to be compromised within hospital settings where a patient (and also the referring general practitioner) might contact many different clinicians.

**Box 4 Priority concerns about place, organisation, and delivery of care and need for new developments**

**Out of hours/continuity of care**

“The area of concern in my practice/geographical area is the provision of care around the clock. During office working hours there is a good provision of services but other than office hours patients don’t get a good service . . . there is no district nurse support for out of hours care. There is minimal specialist palliative care cover but no generic care” (generalist)

**Health and social care interface**

“The division between social services and health care is an absolute nightmare. . . There are huge delays in discharges and there is this artificial divide between whether patients’ needs are health or social care, when in many cases they’re both . . . the speed at which patient assessments are made is too slow, meaning that some patients who may have been able to stay at home end up having to be admitted because they didn’t receive care as quickly as they required” (generalist)

**Access**

“Frail older people and their families, with or without dementia, at the end of their life are regarded as a drain on hospital resources and are not treated in the same way as younger patients with cancer. Similarly older frail people dying in care homes, if they are not part of the GSF [gold standards framework] do not have the same support from the local palliative care teams. This is exacerbated by the difficulty staff have identifying when a patient/resident is dying” (generalist)

**Measuring outcomes**

“Measuring outcomes is extremely difficult within palliative care . . . it is extremely difficult to measure quality . . . palliative care does not restore people to working life and is not economically beneficial to wider society” (generalist)

**End of life care tools**

“LCP [Liverpool care pathway] can work well but [there is] a big challenge in keeping it going with staff turnover” (policy maker)
“The LCP is very good, we are virtually running it in the whole hospital now in a limited way . . . I don’t think it improves care very much without an education programme that goes with it. Not a cheap and easy way . . . very labour and resource intensive” (specialist in palliative care)
“GSF works well but the effectiveness of implementation varies from practice to practice. Similarly the LCP is a very good model to support generalist provision of care for patients in their last days of life” (generalist)
“End of life care initiatives are often not evidence based and/or rigorously evaluated for cost effectiveness” (academic)
“We need more research on the benefits, harms and risks of encouraging advanced care planning prior to the national recommendation of implementation of any particular advance care planning tool” (generalist)

*Interface between health and social care*—Groups reported communication between health and social care as problematic, with the separation of health and social care services thought to be contributing to delays in discharge from hospital. Patients’ needs often crossed over both services, and the process whereby patients were identified as needing priority social support when discharged home was seen to take too long for those whose life expectancy was limited. Participants described patients dying in hospital before a decision on the required level of social care support had been made.

*End of life care tools*—Both generalists and specialists in palliative care responded positively about end of life care tools such as the gold standards framework. There were, however, concerns about the lack of evidence to support their use and development and to justify the investment of time. Within hospital settings and care homes the Liverpool care pathway was thought to be a good model of care, but it was considered difficult to sustain when there was a high turnover of staff and a lack of funds to provide the necessary education.

### Need for new developments

*Prognostic indicators and outcome measurement*—The lack of prognostic indicators and clinical triggers to inform decision making about when end of life care should start was thought to be an important gap in applying generalist end of life care. Measuring effectiveness in end of life care was particularly difficult as patients’ symptoms and quality of life worsened towards death.

*Costs and resources*—The low priority accorded to funding end of life care within the NHS and the reliance on the voluntary sector for hospice care was seen to reflect a major weakness in the system. Many generalists, specialists, and academics (51/167) suggested that good end of life care could not be provided or improved on without increasing overall resources within the NHS, especially for patients with non-malignant diseases. Knowledge was needed urgently about the relative cost effectiveness of care at home in comparison to hospital and hospice care.

*Improving access*—Difficulties in prognosis, particularly in non-malignant disease, were thought to hinder access to appropriate help from generalist and specialist palliative care services. While most people approaching the end of life were elderly, there were concerns that frail older people were treated less comprehensively than younger people and could be seen as a drain on resources. Access to services by specific groups, such as those with cultural and language differences, learning difficulties, and mental health problems, was thought to be problematic, particularly by voluntary and user groups and academics.

*Identifying carers’ needs*—All groups recognised the vital role of informal carers in providing end of life care, and there were concerns that carers’ needs were poorly recorded and understood and that their views and preferences were not adequately taken into account. Improved service provision to support carers was deemed a priority.

### Differences between consultation priorities

There was a high level of agreement between the different consultations in terms of priority issues and topics for research (box 5). These included improving service provision, out of hours care, non-cancer care, place of care and death, and the experiences of patients and carers.

**Box 5 Priorities for research to improve generalist end of life**

**English national organisations (30 voters)**

- Service provision (53%)
- Place of care and death: care at home (50%)
- Service provision: improving access (40%)
- Resources/health economics (37%)
- Patients’ experience (33%)
- Non-cancer: older people (30%)

**London (38 voters)**

- Non-cancer care (42%)
- Patients’ experience (38%)
- Place of care and death: care at home (38%)
- Resources/health economics (38%)
- Service provision: emergency/out of hours care (34%)
- Service provision/models of care (34%)

- Service provision: out of hours (67%)
- Education (63%)
- Place of care and death: care homes (58%)
- Patients’ experience (52%)
- Place of care and death: hospital care (32%)
- Non-cancer care (32%)

**Warwickshire (25 voters)**

- Service provision: out of hours (84%)
- Patients’ experience (52%)
- Education: support needs of generalists (48%)
- Service provision: models of care; cost *v* quality (44%)
- Education and training (44%)
- Generalist role (40%)

**Scotland (55 voters)**

- Service provision: changing practice (58%)
- Non-cancer (55%)
- Patients’/carers’ experience (55%)
- Service provision: out of hours (49%)
- Service provision: inequalities (33%)
- Place of care and death: hospital care (33%)

79.5% (167/210) of participants voted. Each voter cast five votes for priority areas, but not in priority order. Up to 17 categories were identified for each consultation.^33^

The geographically based consultations placed higher priority on improving out of hours care than participants from the English national organisations, perhaps reflecting the greater involvement of generalist practitioners. Access to services based on geographical location was an issue of greater concern in Scotland, where remote, rural, and island communities with widespread populations have less access to hospital, hospices, and specialist end of life care.

## Discussion

### Improving the evidence base

Much needs to be done to support generalists in providing care to patients at the end of life and to their carers. It is surprising that there has been so little research and development, even in the United States, where there is considerable variation in type of care provided by hospitals.^4^ ^35^ The working areas identified by recent working groups (care pathways, service models, commissioning, care homes, quality and outcomes, workforce development, costs) were all issues raised by our participants (www.healthcareforlondon.nhs.uk/background.asp).^36^ These issues were echoed in a workshop held in Canada to build primary care capacity in palliative care.^37^ Lack of funding, shortage of trained professionals, and insufficient training and infrastructure reflect common international problems for generalist end of life care.

The Department of Health’s strategy adopted the term “end of life care” as it was thought to be easily understood by the public and not, like palliative care, associated with cancer.^24^ We found, however, that interpretation of this term and what constituted end of life, palliative, and terminal care varied between participants. Differences in perception might affect when end of life care takes place—for example, after assignment of a poor prognosis or during the last few days of life. Difficulties in prognosis, particularly for non-malignant disease, compound this obstacle and emphasise the need for practical solutions to support recognition of when end of life care should begin. The gold standards framework proposed six criteria for guidance concerning prognosis in major non-malignant conditions. These criteria, however, can be difficult to apply—for example, in advanced chronic obstructive pulmonary disease it is difficult to give an accurate prognosis because even among the most severely affected, mean survival might be two years or more.^38^

The end of life care programme in England focused on supporting generalist models of care such as the gold standards framework and the Liverpool care pathway.^28^ There are concerns, however, about the evidence base of such programmes and the need for further evaluation. While implementation of the gold standards framework has improved aspects of the quality of palliative care, there are variations in the extent to which this has occurred in general practice teams.^39^ Further research is needed to explore the “levers and barriers” to effective general practice based palliative care using the gold standards framework as there are differences in the organisation of general practices that continue to sustain progress with the framework compared with those that no longer hold framework meetings.^40^ The end of life care programme website provides examples of case studies of different interventions and models of care (www.endoflifecareforadults.nhs.uk/eolc/), but there is limited evidence to show how well many of these work and how they could be sustained and transferred to other contexts. More rigorous evaluation of current tools and detailed assessment of resources needed to extend provision of end of life care are required. This might be easier to achieve in England once the baseline review of end of life care services currently being undertaken by primary care trusts is published and more concrete information becomes available about the range of (and need for) current services.^41^ Developing effective outcome measures to improve the evidence base, however, might also depend on understanding more about the different perceptions held about what comprises a good “end of life” and how “end of life care” is defined. While this was beyond the scope of our study, it is clearly an important factor that needs consideration. The need for a greater evidence base is further underpinned by projections that between 2012 and 2030 deaths in the UK will increase by 17% to nearly 590 000 a year and the need for expansion of provision of end of life care in both primary and secondary care services.^42^

### Supporting generalists in providing end of life care

Many of our participants were generalists who described the difficulties of integrating end of life care within a generalist caseload where there were many other competing priorities. They described some generalists as being disengaged from end of life care. While recognising the importance of the generalist workface, the end of life care strategy,^28^ the London review (www.healthcareforlondon.nhs.uk/background.asp) and the report of the Royal College of Physicians have paid little attention to this issue. 30 Much more needs to be known about the checks and balances that shape decision making in service provision at general practice and at primary care organisation level and within acute hospital trusts. A key issue here is ensuring that educational and training budgets in end of life care are ringfenced. Ensuring that sufficient funds are made available to support education and training for generalists would form a major investment in quality care and patient choice, enabling workforce development and uptake of the end of life care tools. This is endorsed by the Department of Health^28^ and Royal College of Physicians,^30^ which recommends that “generic palliative care should be a core part of training and a subject for the continuing professional development of all.”^30^ It is unclear, however, who should provide such education and training, and respiratory and cardiac specialists, for example, might well hold greater expertise in end of life care in these conditions than specialists in palliative medicine.

### Limitations and outcomes of the consultation

Our consultation was part of a scoping exercise with a limited timescale rather than an in-depth qualitative study or complete national survey and as such has inherent limitations in terms of comprehensiveness. We did, however, adopt a rigorous and flexible approach in terms of sampling, data collection, and analysis to enable widespread participation. The research team included a broad range of stakeholders, and we recruited from a wide range of organisations, but we cannot claim to represent all potential stakeholders, particularly users of services, because of our focus on user groups. While the consultation took place in different geographical areas of England and Scotland, we cannot claim that we represent all geographical regions. There was, however, considerable enthusiasm to participate, reflected in the high response rate.

A major outcome of the consultation was the identification of research priorities in generalist end of life care to address gaps in knowledge.^31^ ^32^ ^33^ These research priorities included learning more about good models of care and service provision out of hours, in hospital, among people suffering from non-malignant disease, and among older people, identifying best practice, widening access to care, and understanding more about place of care and death particularly for people with non-malignant disease. The focus of new research should be on the experience of services in patients and carers and the implications on resources and health economics of developing improved services. Box 6 presents examples of research questions suggested as a result of the consultation. The issues and priorities identified in this research should help to inform the Department of Health’s implementation of the end of life care strategy, especially in developing its research programme.

**Box 6 Examples of research questions to improve generalist end of life care arising from the consultation**

**Improving service provision**

- How does end of life care integrate within generalist caseloads?
- How to engage the disengaged generalist
- How to improve access to health and social care out of hours
- Do end of life care tools provide better care, reduce costs, increase choice etc?

**Care for non-cancer patients**

- What models of care work at the end of life?
- How can non-cancer patients be best identified for supportive and palliative care in the community?
- How can non-cancer assessment and planning be best done in the community?

**Place of care and death**

- What are the full costs of keeping a patient at home?
- How can national policies support locally determined delivery of best practice?
- What support do care homes need to prevent emergency admission?

**Experience of patients and carers**

- What do patients want from care providers?
- What is the level of patients’ experience of care we are aiming for?
- What do patients know about what they can access and expect?

### Conclusion

Definitions of end of life care need clarification and standardisation as lack of clarity can hinder access to services. The competing priorities and incentives faced by generalists act as barriers to improving care at the end of life and more needs to be known about the context of provision of end of life care. Access to education and training in care at the end of life is limited for generalists but is essential if they are to develop and maintain their knowledge and skills.

**What is already known on this topic**

Most people are cared for by generalists at the end of life and die in generalist settings
Government policy is promoting initiatives to increase and improve generalist end of life care
Less is known about the provision of generalist end of life care compared with specialist palliative care

**What this study adds**

Practitioners, service commissioners, academics, and representatives of user and voluntary groups do not agree about what constitutes generalist end of life, palliative, and terminal care
Variability of practice, lack of routine educational and training opportunities, and limited resources are concerns
Effective models for patients with non-malignant disease, for out of hours care, and for hospital care need to be identified and trialled
Research priorities should incorporate the perspectives of patients and carers and implications on resources and health economics

## Notes

We are grateful to all individuals participating in this consultation. We thank colleagues for their support including Matthew Hotopf, Geoffrey Mitchell, Naomi Fulop, Marilyn Kendall, David Chinn, Fliss Murtagh, Barbara Gomes, Sue Hall, Elizabeth Summers, Dan Munday, Clare Bawden and staff at the King’s Fund, Jenny Lunan, and Emma Camplejohn. We also thank Genevieve Casey, Stephen Peckham, Damian O’Boyle, and Emma Hawkridge at the National Institute of Health Research Service Development and Organisation.

Contributors: IJH was lead applicant in the grant application, supported by CS. MG, PW, AW, SAM, SB, SF, JS, JD, SD, AR, and Matthew Hotopf contributed to the study design. CS coordinated the study, supported by IJH and PW. CS led the London and National components of the study supported by PW, IJH, and SD; MP and SW undertook data collection. AW and SAM undertook data collection and analysis for the Scottish component, SF and SB for the East of England, and JS and JD for the Warwickshire components of the study. CS led central analysis with contributions from IJH, PW, MG, AW, SAM, SB, SF, JS, JD, SD, MP, SW, AR, Matthew Hotopf, JK, and KL. CS, IJH, and PW drafted and revised the manuscript with contributions from all authors. IJH and CS are guarantors.

Ethical approval: King’s College London research ethics committee (CREC/06/07-22).

Provenance and peer review: Not commissioned; externally peer reviewed.

## Notes

**Cite this as: ***BMJ* 2008;337:a1720
